# The Consumption of Dairy and Its Association with Nutritional Status in the South East Asian Nutrition Surveys (SEANUTS)

**DOI:** 10.3390/nu10060759

**Published:** 2018-06-13

**Authors:** Khanh Le Nguyen Bao, Sandjaja Sandjaja, Bee Koon Poh, Nipa Rojroongwasinkul, Chinh Nguyen Huu, Edith Sumedi, Jamil Nor Aini, Sayamon Senaprom, Paul Deurenberg, Marjolijn Bragt, Ilse Khouw, on behalf of the SEANUTS Study Group

**Affiliations:** 1 National Institute of Nutrition, 48B Tang Bat Ho Street, Hanoi 10000, Vietnam; bkhanhnin@gmail.com (K.L.N.B.); nguyenhuuchinhvdd@gmail.com (C.N.H.); 2 Persatuan Ahli Gizi Indonesia, Jalan Hang Jebat III/F3, Kebayoran Baru, Jakarta 12120, Indonesia; san_gizi@yahoo.com (S.S.); edith.sumedi@yahoo.com (E.S.); 3 Universiti Kebangsaan Malaysia, Jalan Raja Muda Abdul Aziz, 50300 Kuala Lumpur, Malaysia; pbkoon@ukm.edu.my (B.K.P.); ainijamil@ukm.edu.my (J.N.A.); 4 Institute of Nutrition Mahidol University, Phuttamonthon 4, Nakhon Pathom 73170, Thailand; nipa.roj@mahidol.ac.th (N.R.); sayamon.sen@mahidol.ac.th (S.S.); 5 Nutrition Consultant, 055 Laurel Street, 3319 Ramon, Isabela, The Philippines; paul.deurenberg@gmail.com; 6 FrieslandCampina, Stationsplein 4, 3818 LE Amersfoort, The Netherlands; marjolijn.bragt@frieslandcampina.com

**Keywords:** dairy, SEANUTS, stunting, underweight, vitamin A, vitamin D, haemoglobin

## Abstract

Despite a major decrease in undernutrition worldwide over the last 25 years, underweight and stunting in children still persist as public health issues especially in Africa and Asia. Adequate nutrition is one of the key factors for healthy growth and development of children. In this study, the associations between dairy consumption and nutritional status in the South East Asian Nutrition Survey (SEANUTS) were investigated. National representative data of 12,376 children in Indonesia, Malaysia, Thailand, and Vietnam aged between 1 and 12 years were pooled, representing nearly 88 million children in this age category. It was found that the prevalence of stunting and underweight was lower in children who consumed dairy on a daily basis (10.0% and 12.0%, respectively) compared to children who did not use dairy (21.4% and 18.0%, respectively) (*p* < 0.05). The prevalence of vitamin A deficiency and vitamin D insufficiency was lower in the group of dairy users (3.9% and 39.4%, respectively) compared to non-dairy consumers (7.5% and 53.8%, respectively) (*p* < 0.05). This study suggests that dairy as part of a daily diet plays an important role in growth and supports a healthy vitamin A and vitamin D status.

## 1. Introduction

Although the proportion of under nutrition in the Southeast Asian region decreased tremendously from 31% in 1990 to 10% in 2015, 16% of the children under 5 years of age are still moderately to severely underweight [1]. Moreover, it is projected that more than 25% or 165 million children under 5 years of age are stunted, of which 90% reside in Africa and Asia [2].

In line with these numbers, the South East Asian Nutrition Surveys (SEANUTS) also showed that under nutrition is still a major issue in the four countries of Indonesia, Malaysia, Thailand, and Vietnam, yet at different degrees [3,4,5,6]. Overall, the prevalence of stunting in children 0.5–12 years of age in these four countries was around 15%, with rural Indonesia having the highest prevalence (38.8%) and urban Thailand the lowest prevalence (4.2%). The prevalence of underweight varied between 6.4% for urban Thai children and 28.9% for rural Indonesian children. Moreover, SEANUTS also found a high prevalence of vitamin D insufficiency in all of the four countries, varying from 20% in Thailand up to 44% in Malaysia [7].

Nutrition is an important factor for healthy growth and development in children. Dietary guidelines recommend a well-balanced diet including all major food groups for sufficient intake of necessary macro- and micronutrients [8]. However, SEANUTS showed that a large proportion of children did not meet their daily recommended intake (RDI) of many nutrients including calcium, iron, vitamin C, and vitamin D [3,4,5,6]. It was also found that with the exception of meat/poultry, Malaysian children did not meet the recommendations of daily intake for each food group [9].

Dairy products contribute to a healthy diet by providing energy, protein, and micronutrients such as calcium, magnesium, and vitamins B1, B2, and B12 [10]. Dairy protein is considered to be of high quality as it provides all the essential amino acids, with high bioavailability [11]. In many recommendation and guidelines, dairy is often advised as part of a healthy diet [12]. Analyses of SEANUTS data of Vietnam and Indonesia showed that children consuming dairy products were better in achieving RDI levels for protein, calcium, iron, zinc, vitamins A, B1, B2, B3, C, and D, compared to children who did not consume dairy [13,14].

A few studies have linked dairy consumption with nutritional status [12,15], but many of them were relatively small or conducted in selected groups. The objective of this SEANUTS analysis was to determine whether children who consumed dairy products as part of their daily diet had a better nutritional status as measured by anthropometric indices and blood status for iron, vitamin A and D compared to children who did not.

## 2. Materials and Methods

SEANUTS is a nationally-representative multi-centre survey in Indonesia, Malaysia, Thailand, and Vietnam, conducted between 2010 and 2011, to assess the nutritional status and lifestyle factors of over 16,500 children aged 0.5–12 years old. A multi-stage cluster sampling, stratified for geographical location, gender and age was carried out. The survey was conducted according to the guidelines laid down in the Declaration of Helsinki and in each country the survey and its procedures were approved by local ethical committees. The survey is registered in the Netherlands Trial Register as NTR2462. Details of SEANUTS methodology are described elsewhere [3,4,5,6,16]. In the current study only data of children older than 1 year were used.

Nutrient intake and dairy consumption were calculated from 24 h recall questionnaires (Indonesia, Thailand, and Vietnam) and/or from semi-quantitative food frequency questionnaires (FFQ, Indonesia, Malaysia, Thailand) using the local food composition tables.

Even though different local dietary guidelines advice children to consume 2–3 servings of dairy per day (500–750 g dairy), many children in South East Asia do not meet this recommendation. Therefore, we decided to use lower criteria. A child was defined as ‘dairy user’ if he/she consumed a minimum of either 15 g milk powder (equals about 100 mL milk when dissolved in water), 100 g UHT/flavoured/cultured milk or drink yoghurt, 50 g yoghurt, 10 g condensed milk or ice cream, or 5 g cheese on a daily basis. Each of these amounts was also considered to reflect one dairy consumption. If the consumption of each individual dairy product was below these criteria but the sum of all dairy products exceeded 100 g, the child was classified as ‘dairy user’ as well. If different dairy products were consumed in the above mentioned minimum amounts, consumption would be categorized as ≥2 consumptions per day.

Information on the level of education of the child’s mother and family income was collected from the parents or primary caregivers using a structured questionnaire. Income was categorized into country specific quintiles, while education was classified into three groups as primary school or lower, secondary education or tertiary education.

Weight was measured using calibrated digital scales accurate to 0.1 kg. Height was measured using wall-mounted stadiometers accurate to 0.1 cm. Body mass index (BMI, kg/m^2^) was calculated as weight divided by height squared. Weight for age Z-scores (WAZ), BMI for age Z-scores (BAZ) and height for age Z-scores (HAZ) were calculated based on WHO references [17,18]. Children with HAZ, WAZ and BAZ lower than −2 SD of the reference value were classified as stunted, underweight and thin, respectively. The cut-off values for overweight and obesity among children <5 years were +2 SD and +3 SD, whereas they were +1 SD and +2 SD, respectively, for children aged ≥5 years.

Blood from a subsample of subjects was analysed for haemoglobin (Hb), ferritin, vitamin A (serum retinol), and vitamin D (25-hydroxyvitamin D (25(OH)D)). Details of the methodologies used are described in Schaafsma et al. [16]. Anaemia was defined as Hb concentrations <110 g/L for subjects <5 years, <115 g/L for subjects aged 5–11.9 years, and <120 g/L for subjects aged ≥12 years [19]. Iron deficiency was defined as serum ferritin concentrations <12 μg/L for children aged <5 years and <15 μg/L for children aged ≥5 years [20]. Serum retinol concentration <0.70 μmol/L was used as an indicator for vitamin A deficiency [21], whereas circulating 25(OH)D concentration <50 nmol/L was used as an indication for vitamin D insufficiency [22].

Data were weighted using age, gender and residence weight factors to extrapolate to the total population per country. The weight factors were based on data from the relevant national Statistical Offices. In the pooled analyses, the population size of each country was taken into account to avoid bias due to the effect of larger population number.

Data were analysed using SPSS version 20.0 (IBM Cooperation 2011, Armonk, NY, USA) with complex sample techniques. Differences between groups were tested using analysis of (co)variance after correction for possible confounders. Differences in categorical variables across groups were tested using logistic regression with correction for confounding variables (age, residence, mother’s educational level, and income level). Values are expressed as mean and SE and level of significance is set at *p* < 0.05.

## 3. Results

The total sample size in the present analyses was 12,376, representing nearly 88 million children. Table 1 shows the anthropometric characteristics of the children and their blood profiles.

Overall, 68% of the children consumed dairy (Table 2) according to the criteria as defined in the Materials and Methods, although it varied per country. Most of the children in Thailand (98%) consumed dairy on a daily basis, followed by Malaysia (69%), Indonesia (52%), and Vietnam (47%), respectively. For the types of dairy product, amongst the four countries, UHT/flavoured milk was the most consumed (32%), followed by powder milk (22%), but the consumptions varied across countries. In Malaysia, mostly powder milk was consumed (41%). In Thailand and Vietnam, the children drank mainly UHT/flavoured milk (84% and 34%, respectively). In Indonesia mainly condensed milk was consumed (27%). Yoghurt and cheese were dairy formats less consumed by the children (data not shown).

There were less dairy users in the older children (from approx. 6 years onwards), except for Thailand where almost 100% of the children were dairy consumers irrespective of their age. Dairy consumption did not differ between boys and girls, but in Indonesia and Vietnam more urban children (62% and 72%, respectively) consumed dairy compared to rural children (44% and 34%, respectively, *p* < 0.05). The consumption was also dependent on maternal education level and socio-economic status except for Thailand. Significant more children consumed dairy when their mothers were higher educated and when their families were in the higher income quintiles (data not shown).

Some 42.6% of the children were categorized in the <1 dairy consumption per day group, while 39.3% and 18.1% had 1 and ≥2 dairy consumptions per day, respectively. Mean dairy intake in the group of 1 dairy consumption per day was 281 ± 6 g/day and in the group of ≥2 dairy consumptions per day 521 ± 6 g/day. Children in the groups of 1 or ≥2 dairy consumptions per day had higher total dietary intakes compared to children consuming <1 dairy consumption per day when compared to the different local recommended dietary allowances of each country (Table 3).

In Table 4 the mean (SE) of anthropometric variables in the three dairy user groups and the prevalence for malnutrition is shown. Weight, height, WAZ, and HAZ were significantly higher in children in the groups of 1 or ≥2 dairy consumptions per day compared to children who consumed <1 dairy consumption per day. Consequently, the prevalence for underweight and stunting was lower in the children drinking more than 1 or ≥2 dairy consumptions per day. For height, HAZ, WAZ, and stunting, there were also significant differences between 1 and ≥2 dairy consumptions per day. However, BMI, BAZ, or the prevalence for thinness, overweight, and obesity did not differ between the three groups.

One or ≥2 consumption of dairy per day was associated with a lower risk of being stunted and children who had ≥2 dairy consumptions per day were less likely to be underweight (Table 5). Dairy consumption was not significantly associated with the risk of being thin, overweight or obese (Table 5).

In Table 6, the sample sizes, mean blood values and prevalence are shown for anaemia, iron deficiency, vitamin A deficiency, and vitamin D insufficiency. One or ≥2 dairy consumptions per day were neither associated with the prevalence of anaemia which was around 11%–13% in the three groups nor with the prevalence of iron deficiency, with or without correction for inflammation even though there is a trend for a lower prevalence in the 1 and ≥2 dairy consumption groups. In contrast, the prevalence of vitamin A deficiency and vitamin D insufficiency were significant lower in children drinking 1 or ≥2 dairy consumptions per day. For vitamin A deficiency, the prevalence in the group of 1 dairy consumption per day was 3.9% while it was 7.5% in the group of <1 dairy consumption per day. For vitamin D insufficiency, the prevalence was 39.4% and 53.8%, respectively. Consequently, the odds of being vitamin A deficient or vitamin D insufficient were lower in the groups of 1 or ≥2 dairy consumptions per day.

## 4. Discussion

The present study shows that incidence of dairy consumption was positively associated with the nutritional status of 1–12-year-old children in the SEANUTS population of Indonesia, Malaysia, Thailand, and Vietnam based on anthropometric indices. Children were less likely to be stunted or underweight when dairy was part of their daily diet. Stunting is associated with increased morbidity and impacts cognitive development [23,24]. It is also a risk factor for chronic diseases in adulthood [25].

Previous intervention and observational studies have shown a positive effect of milk and dairy products on the growth of preschool and school-aged children [26,27,28,29]. As early as 1928, it was reported that milk supplementation in 5–14-year-old Scottish children resulted in approximately 20% more height gain compared to children who received a biscuit as control [26]. Another study that followed the effect of a school milk programme in 6–9-year-old Malaysian primary school children showed that there was a reduction in underweight, stunting, and wasting after two years [27]. In Indonesia, it was shown that supplementation of fortified milk to 6–59 month old children was associated with a lower risk of stunting [29]. A meta-analysis of 12 trials examining the association between dairy consumption and height showed that supplementation of approximately 245 mL milk on a daily basis resulted in an additional 0.4 cm growth per year [28]. Our current results confirm the positive association between dairy consumption and growth.

Recently, it was hypothesized that insufficiency in essential amino acids may be an important limiting factor in linear growth [30]. When specific amino acids, especially the essential ones, are deficient in the children’s diet, protein and lipid synthesis and cellular growth is negatively affected [31]. Dairy is known to provide all essential amino acids. Moreover, dairy protein has a high protein-digestibility-corrected amino acid score (PDCAAS) [12] indicating that the amino acids are easily bioavailable for digestion, absorption, and utilization by the body. This may be the mechanism behind the association between dairy consumption and lower prevalence and risk of stunting. Further analyses of the dietary intake data of SEANUTS showed that stunted children had a lower intake of total protein compared to normal height children (data not shown). Moreover, intake of animal protein was also lower (data not shown), which is also linked to stunting [32,33].

The association between dairy and overweight/obesity is more complicated than dairy and linear growth. A systematic review of 19 studies in both children and adults by Louie et al. [34] showed results ranging from dairy having a protective effect against weight gain (nine studies), to no impact (seven studies), or to even increasing the risk of weight gain (three studies) and the authors, therefore, stated that it is difficult making firm conclusions. Our data showed no difference in overweight/obesity prevalence between dairy consumers and non-dairy consumers and also the odds for being overweight or obese did not differ between the three groups.

The prevalence of vitamin A deficiency and vitamin D insufficiency in the group of 1 or ≥ 2 dairy consumptions per day was lower than in the group with <1 dairy consumption per day. Moreover, the former were also found to be less likely vitamin A or vitamin D deficient (Table 6). A possible explanation could be that dairy consumption is associated with healthier food choices and better total diet quality in general [35] resulting in better intakes of vitamin A and D. On the other hand, although cow’s milk does not naturally contain high levels of these vitamins, today’s trend is that many dairy products in Indonesia, Malaysia, Thailand, and Vietnam, especially those in powder formats and those targeted for children, are often fortified with micronutrients such as vitamin A, B2, and D. Vitamin D insufficiency is a major issue in many countries, also in the SEANUTS countries, despite the abundance of sun light available in the Southeast Asian region [7,36,37]. Therefore, dietary vitamin D intake, either via foods naturally rich in vitamin D such as oily fish and eggs, or via vitamin D fortified foods, might become more and more important. The relatively high percentages of vitamin D insufficiency in the groups of dairy consumers may warrant evaluation of fortification policies and practices by government and manufacturers [38].

No significant differences were found in anaemia and iron deficiency between the three groups, which is not surprising as dairy is not a good dietary source for iron. In contrast, a trial in Vietnam showed a decrease in anaemia prevalence from 46.7% to 9.3% and from 43.7% to 19.2% in the 7–8-year-old children after 6 months of consuming fortified milk and regular milk, respectively [39]. A reason for these different findings could be that the anaemia prevalence in the SEANUTS cohort was much lower than in the children studied by Lien et al. [39]. Another reason could be the type of milk consumed in the present study, whereby UHT milk was the format most commonly consumed, which is normally not iron-fortified while powder milk and especially growing up milk powder are fortified with iron.

Although the prevalence of lactose intolerance is higher in the Asian region compared to for example Western Europe, it is not a clinical issue in younger children. For adults and older children, most who have been diagnosed with lactase deficiency can often tolerate consuming some dairy products. Dairy formats such as yoghurt can be an alternative for normal milk products as fermented milk products are better tolerated by individuals who are lactose-intolerant [12,40]. For this reason, the possible higher risk of being lactose intolerant should not be a contra-indication for consuming dairy as prevention for stunting or micronutrient deficiencies.

The current study has its limitations as it was not designed to study the relationship between nutritional status and dairy consumption. Also, different methodologies were used to assess dairy consumption. Furthermore, local food composition databases might not be up to date in a fast changing market of dairy products and may differ between countries.

## 5. Conclusions

In conclusion, the results showed that children who consumed dairy were less likely to be stunted or underweight and less likely to be vitamin A deficient or vitamin D insufficient. Future studies should focus on the status of other micronutrients like B vitamins or zinc as more and more data are emerging that those deficiencies are prevalent in the Asian region [41,42]. On the other hand, more intervention studies should be conducted for a better understanding of the role of dairy and how to improve the nutritional status of children. Then, in relation to public health, national policies should consider the availability and accessibility of dairy to its population to support the healthy growth and development of children.

## Figures and Tables

**Table 1:** Characteristics of the children (1–12 years old) per country and in the four countries combined.

	Indonesia		Malaysia		Thailand		Vietnam		All Four Countries	
**Sample size**	3163		3472		2943		2798		12,376	
**Population**	53,184,526		5,740,266		9,662,074		19,402,735		87,989,601	
	**Mean**	**SE**	**Mean**	**SE**	**Mean**	**SE**	**Mean**	**SE**	**Mean**	**SE**
Age (years)	6.6	0.1	7.1	0.1	7.2	0.1	7.4	0.1	7.1	0.0
Weight (kg)	19.4	0.2	25.5	0.4	24.3	0.3	22.1	0.2	22.9	0.1
Height (cm)	110.5	0.4	118.2	0.6	118.2	0.5	118.0	0.3	116.2	0.2
BMI (kg/m^2^)	15.4	0.1	17.0	0.1	16.4	0.1	15.4	0.0	16.1	0.0
HAZ	−1.44	0.03	−0.55	0.03	−0.52	0.02	−0.88	0.02	−0.84	0.01
WAZ	−1.23	0.04	−0.33	0.03	−0.39	0.03	−0.81	0.03	−0.70	0.02
BAZ	−0.47	0.03	0.14	0.03	−0.10	0.03	−0.54	0.03	−0.23	0.02
Hb (g/L)	122	0	131	0	125	1	127	1	126	0
Ferritin (μg/L)	46.8	1.0	48.5	1.5	59.4	1.9	49.1	2.3	51.0	0.9
Retinol (μmol/L)	1.47	0.02	1.06	0.01	1.23	0.02	1.05	0.03	1.18	0.01
25-hydroxyvitamin D (nmol/L)	53.1	0.9	52.7	1.0	59.5	1.1	55.9	1.7	55.7	0.7

BMI: body mass index; HAZ: height for age Z-score; WAZ: weight for age Z-score; BAZ: body mass index for age Z-score, Hb: Haemoglobin. Recommended values for Hb are >110 g/L for subjects <5 years, >115 g/L for subjects aged 5–11.9 years and >120 g/L for subjects aged ≥12 years. Recommended iron values are >12 µg/L for children aged <5 years and >15 µg/L for children aged ≥5 years. Recommended values for retinol is >70 µmol/L and for vitamin D >50 nmol/L.

**Table 2:** Percentage of children consuming different dairy products and percentage of dairy users per country and for the four countries combined.

	Indonesia	Malaysia	Thailand	Vietnam	All Four Countries
UHT/flavoured milk (>100 g)	5	3	84	34	32
Milk powder (>15 g)	19	41	14	11	22
Condensed milk (>10 g)	27	15	0	4	12
Dairy user *	52	69	98	47	68

* Dairy user is defined as a minimum average consumption of either 15 g powder milk, 100 g UHT/flavoured milk, 50 g yoghurt, 10 g condensed milk or ice cream, or 5 g cheese on a daily basis. In case the consumption of each individual dairy product was below these criteria but the sum of all dairy products exceeded 100 g, the child was classified as ‘dairy user’ as well.

**Table 3:** Total dietary intake as percent of local RDA for the three different dairy user groups.

Dairy Per Day	<1 Dairy Consumption		1 Dairy Consumption		≥2 Dairy Consumptions	
	Mean	SE	Mean	SE	Mean	SE
Energy	74 ^a^	1	90 ^b^	1	97 ^c^	1
Protein	119 ^a^	1	158 ^b^	1	179 ^c^	1
Calcium	55 ^a^	1	96 ^b^	1	83 ^c^	1
Iron	76 ^a^	1	117 ^b^	2	101 ^b^	2
Zinc	75 ^a^	1	112 ^b^	1	148 ^c^	2
Vitamin B1	71 ^a^	1	114 ^b^	1	141 ^c^	2
Vitamin B2	69 ^a^	1	147 ^b^	2	184 ^c^	2
Vitamin B3	63 ^a^	1	92 ^b^	1	103 ^c^	1
Vitamin C	73 ^a^	2	132 ^b^	3	99 ^c^	2
Vitamin A	63 ^a^	1	96 ^b^	1	88 ^c^	2
Vitamin D	41 ^a^	1	86 ^b^	2	101 ^c^	2

^a,b,c^: Different letters in superscripts indicate significant difference with other cells in the same row.

**Table 4:** Anthropometric variables in the different dairy consumption groups and prevalence of malnutrition after corrections for confounders †.

	<1 Dairy Consumption/Day		1 Dairy Consumption/Day		≥2 Dairy Consumptions/Day	
	Mean	SE	Mean	SE	Mean	SE
Age (years)	7.8 ^a^	0.1	6.3 ^b^	0.1	7.1 ^c^	0.1
Height (cm)	114.6 ^a^	0.1	115.9 ^b^	0.1	117.1 ^c^	0.1
Weight (kg)	22.2 ^a^	0.2	22.8 ^b^	0.1	23.2 ^b^	0.2
BMI (kg/m^2^)	16.0 ^a^	0.1	16.0 ^a^	0.1	16.1 ^a^	0.1
HAZ	−1.09 ^a^	0.02	−0.84 ^b^	0.02	−0.63 ^c^	0.02
WAZ	−0.90 ^a^	0.03	−0.73 ^b^	0.03	−0.50 ^c^	0.03
BAZ	−0.26 ^a^	0.03	−0.25 ^a^	0.03	−0.17 ^a^	0.03
Stunted (%)	21.4 ^a^		15.2 ^b^		10.0 ^c^	
Underweight (%)	18.0 ^a^		15.0 ^ab^		12.0 ^b^	
Thinness (%)	6.9 ^a^		8.3 ^a^		7.9 ^a^	
Overweight (%)	6.7 ^a^		6.7 ^a^		7.9 ^a^	
Obese (%)	7.3 ^a^		6.9 ^a^		7.3 ^a^	

† Except for age, all values are corrected for differences in age, sex, residence, education level of the mother, income quintile and country. Stunted is defined as HAZ <−2 SD; underweight as WAZ <−2 SD, and thinness as BAZ <−2 SD. Overweight and obesity are defined as BAZ > +2 SD and +3 SD, respectively, for children aged <5 years and >+1 SD and +2 SD, respectively, for children aged ≥5 years. ^a,b,c^: Different letters in superscripts indicate significant difference with other cells in the same row.

**Table 5:** Odds ratio (OR) with 95% confidence interval (CI) for being stunted, underweight, thin, overweight or obese in relation to dairy consumption category.

	Stunted		Underweight		Thinness †		Overweight †		Obese †	
	OR	95% CI	OR	95% CI	OR	95% CI	OR	95% CI	OR	95% CI
<1 dairy consumption/day	1	-	1	-	1	-	1	-	1	-
1 dairy consumption/day	0.7 *	0.6, 0.9	0.9	0.7, 1.1	1.3	1.0, 1.6	1.0	0.8, 1.2	0.9	0.7, 1.2
≥2 dairy consumptions/day	0.5 *	0.4, 0.6	0.7 *	0.6, 0.9	1.2	0.9, 1.6	1.2	1.0, 1.6	1.1	0.8, 1.4

Data are corrected for confounding effect of age, sex, urban/rural, education of mother, income quintile, energy intake and country. Stunted is defined as HAZ <−2 SD; underweight as WAZ <−2 SD, and thinness as BAZ <−2 SD. Overweight and obesity are defined as BAZ > +2SD and +3 SD, respectively, for children aged <5 years and >+1 SD and +2 SD, respectively, for children aged ≥5 years. † Reference category is ‘normal weight’. * *p* < 0.05.

**Table 6:** Mean blood values and prevalence of anaemia, iron deficiency, vitamin A deficiency, and vitamin D insufficiency in the different dairy consumption groups and odds ratios for having a micronutrient deficiency.

	*n*	<1 Dairy Consumption/Day		1 Dairy Consumption/Day		≥2 Dairy Consumptions/Day	
		Mean	SE	Mean	SE	Mean	SE
Hb (g/L)	4149	126 ^a^	0	126 ^a^	0	127 ^a^	0
Ferritin (μg/L)	3041	48.5 ^a^	1.6	50.2 ^ab^	1.5	55.1 ^b^	1.4
Ferritin (μg/L) †	2861	45.7 ^a^	1.4	46.4 ^a^	1.4	49.6 ^a^	1.5
Retinol (μmol/L)	3024	1.15 ^a^	0.02	1.19 ^a^	0.02	1.22 ^a^	0.02
25 Hydroxyvitamin D (nmol/L)	1987	51.6 ^a^	1.3	58.7 ^b^	1.2	57.0 ^b^	0.9
Anaemia		13.1 ^a^		11.6 ^a^		10.9 ^a^	
Iron deficiency †		6.6 ^a^		4.9 ^a^		4.1 ^a^	
Vitamin A deficiency		7.5 ^a^		3.9 ^b^		2.9 ^b^	
Vitamin D insufficiency		53.8 ^a^		39.4 ^b^		40.6 ^b^	
		ODDS	95% CI	ODDS	95% CI	ODDS	95% CI
Anaemia		1	-	0.8	0.6, 1.2	0.8	0.5, 1.1
Iron deficiency †		1	-	0.7	0.4, 1.3	0.6	0.3, 1.2
Vitamin A deficiency		1	-	0.5 *	0.3, 0.9	0.4 *	0.2, 0.7
Vitamin D deficiency		1	-	0.5 *	0.4, 0.7	0.6 *	0.4, 0.8

Hb: haemoglobin. Anaemia is defined as Hb concentrations <110 g/L for subjects <5 years, <115 g/L for subjects aged 5–11.9 years, and <120 g/L for subjects aged ≥12 years. Iron deficiency is defined as serum ferritin concentrations <12 µg/L for children <5 years and <15 µg/L for children ≥5 years. Serum retinol concentration <0.70 µmol/L is an indicator for vitamin A deficiency, whereas 25(OH)D concentration <50 nmol/L was used as an indicator for vitamin D insufficiency. † After correction for inflammation. ^a,b^ Different letters in superscript indicate significant difference with other cells in the same row. * *p* < 0.05. Data are corrected for the confounding effects of age, sex, and residence.
